# Introduction to Special Issue: A New Paradigm of Gene Therapy

**DOI:** 10.3390/pharmaceutics8010001

**Published:** 2016-01-05

**Authors:** Keiji Itaka

**Affiliations:** Laboratory of Clinical Biotechnology, Center for Disease Biology and Integrative Medicine, Graduate School of Medicine, The University of Tokyo, 7-3-1 Hongo, Bunkyo-ku, Tokyo 113-0033, Japan; itaka-ort@umin.net; Tel.: +81-3-5841-0591; Fax: +81-3-5841-1419

Gene therapy is defined as introducing genetic information for therapeutic purposes. Besides the conventional strategy of protein replacement for congenital gene defects, gene therapy may have wide applications, including vaccination against cancer and infectious diseases, regenerative medicine by *in situ* cell regulation by introducing “therapeutic” gene(s), and the ultimate goal of “gene” therapy by gene editing using ZFN, TALENs, and CRISPR–Cas9 technology. In addition, cell therapy combined with *ex vivo* gene introduction is also a promising field.

This Special Issue of *Pharmaceutics* on a “*New Paradigm of Gene Therapy*” addresses the diverse areas related to gene therapy. We greatly appreciate the 13 articles covering various topics. It is interesting that the papers are from many research fields of medicine, pharmaceutical science, and engineering, clearly representing the necessity of integrating many approaches.

The first issue for realizing gene therapy is the preparation of nucleic acids (DNA, RNA) for regulating their intracellular behavior. It is reasonable that the size of DNA is strongly correlated with nuclear entry [1]. For mRNA delivery, the control of immunogenicity is critical, and the many protocols of nucleic acid modification were analyzed, proposing a criterion to determine the modification conditions [2].

The delivery systems of nucleic acids are particularly important, and in this issue we have many studies related to this issue using cationic lipids [3,4], polymers [5,6,7], and functional peptides [8,9]. As is well known, there is a long history of developing effective delivery systems from various fields, and I believe that no single solution will solve all the problems. However, we should have a variety of techniques, because the optimal systems will vary from target to target, and from disease to disease.

It is also appreciated that some studies are challenging experiments for animal disease models including cancer [7,10] and skin disease [8]. Gene therapy is potentially applicable for any kind of diseases, and we hope we will have many applications in the future.

Finally, a unique method of gene delivery, hydrodynamic injection into the liver, is investigated by three papers [11,12,13]. Although the hydrodynamic injection technique is likely applicable for limited organs such as liver and skeletal muscle, it can be available for clinical purpose, and the perspectives are well summarized in the review [13].

A key point of gene therapy is that it has the potential for personalized medicine with relatively low cost, because any kind of protein can be delivered just by changing the DNA sequence. It is also useful that we can directly use biological molecules, such as signaling proteins, for therapeutic purposes, rather than spending significant time and resources developing new drugs to target those molecules. I believe gene therapy will open up new possibilities for various diseases, and hope this special issue will be helpful for overviewing the exciting scientific field of gene therapy.
